# Epithelial to mesenchymal transition is mediated by both TGF-β canonical and non-canonical signaling during axolotl limb regeneration

**DOI:** 10.1038/s41598-018-38171-5

**Published:** 2019-02-04

**Authors:** Fadi Sader, Jean-François Denis, Hamza Laref, Stéphane Roy

**Affiliations:** 1 Department of Biochemistry and Molecular Medicine, Faculty of Medicine, Université de Montréal, Montréal (Québec), Canada; 2 Department of Stomatology, Faculty of Dentistry, Université de Montréal, Montréal (Québec), Canada

**Keywords:** Extracellular signalling molecules, Epithelial-mesenchymal transition

## Abstract

Axolotls have the amazing ability to regenerate. When compared to humans, axolotls display a very fast wound closure, no scarring and are capable to replace lost appendages perfectly. Understanding the signaling mechanism leading to this perfect healing is a key step to help develop regenerative treatments for humans. In this paper, we studied cellular pathways leading to axolotl limb regeneration. We focus on the wound closure phase where keratinocytes migrate to close the lesion site and how epithelial to mesenchymal transitions are involved in this process. We observe a correlation between wound closure and EMT marker expression. Functional analyses using pharmacological inhibitors showed that the TGF-β/SMAD (canonical) and the TGF-β/p38/JNK (non-canonical) pathways play a role in the rate to which the keratinocytes can migrate. When we treat the animals with a combination of inhibitors blocking both canonical and non-canonical TGF-β pathways, it greatly reduced the rate of wound closure and had significant effects on certain known EMT genes.

## Introduction

Regenerative medicine aims to make human regeneration a reality and it has become a key part of biomedical research in the last 25 years. Many lines of enquiry are used in labs around the world to achieve or stimulate regeneration in humans. While research on stem cells takes an important part in the field, the study of regenerating animal models also represents a great avenue to understand how nature has achieved the ability to replace loss body parts or organs. Many animal models, from invertebrates to vertebrates, are capable of a certain amount of regeneration. Which animal model to study depends in great part on the question asked? Urodele amphibians (salamanders) are vertebrates that have the best regenerating abilities amongst tetrapods. They are also the closest phylogenetically to humans with such abilities making them great models for studying regeneration^1^. In this study, the axolotl (*Ambystoma Mexicanum*) limb was used as a model to try and understand how regeneration proceeds in this animal. Many signaling pathways have been shown to be important for regeneration (TGF-β, BMP, p53, Wnt) but we are still in need of many answers before we can hope to fully understand this complex process^2–5^.

Axolotl limb regeneration is morphologically well characterized and separated in different clearly identifiable stages^6,7^. The process can be broken into in two major phases: the preparation phase in which the wound is closed and the blastema is formed; and the redevelopment phase where the limb structures reappear^8,9^. Following an amputation, keratinocytes quickly migrate to close the wound. Cells from the dermis and muscle dedifferentiate, migrate under the wound epidermis and proliferate to form the blastema. The proliferation of the blastema eventually transitions to the start of the second phase of regeneration: the redevelopment phase. The redevelopment phase is characterized by redifferentiation of the different cell types and the patterning of the new limb^7,8,10^.

In the present study, we wanted to assess whether the cellular process known as epithelial to mesenchymal transition (EMT) was involved in limb regeneration. EMTs are known to be involved in mammalian wound healing, development and cancer^11,12^. Interestingly limb regeneration begins with a wound healing phase (preparation phase), then goes through a proliferative state (which could be compared to cancer in some ways) and then finally goes in a phase of redevelopment, thus, making regeneration comparable to development. For each process mentioned, wound healing, cancer and development, many types of EMT have been described^13^. These transitions are described by a change in cellular behavior and shape of epithelial cells which are usually not mobile. Epithelial cells are polarized cell and during EMT they acquire a more elongated shape resembling a mesenchymal cell. The process is reversible, hence the term “transition”. EMTs are usually characterized with the regulation of the expression of different molecular markers^12^. The process can be observed experimentally by looking at the expression of typical markers (Snail, ZEB, Twist etc.), when some mesenchymal markers are upregulated in epithelial cells and epithelial markers are down-regulated (hence epithelial to mesenchymal transition). Expression of genes such as Snails, Twists or ZEBs is up-reregulated during the EMT process. These transcription factors are associated with migration as they induced the expression of proteins like Vimentin and N-Cadherin. They also have a role in inhibiting expression of epithelial markers, such as E-Cadherin^12^.

EMTs are known to be regulated by many signaling pathways^12^. The TGF-β pathway is amongst the most studied one associated with these transitions. Through its canonical (SMAD signaling) and non-canonical (p38 and JNK) pathways, TGF-β has a regulatory effect on EMTs^12,14,15^. The TGF-β canonical pathway signals through the phosphorylation of the TGF-β receptor type II (TβRII) when the ligand (TGF-β1, 2 or 3) binds to it. This binding lead to the dimerization with the TGF-β receptor type I (TβRI). TβRII phosphorylates TβRI which transduces the signal via the phosphorylated SMADs (SMAD2/SMAD3) on their C-Terminus region and participates to the transcription of target genes^16^. Non-canonical pathways can also be triggered when the TβRI/II complex activates certain MAPK’s pathways. The mechanism has not been studied as extensively as the canonical pathway; however, some studies have started to unravel how TGF-β can activate those pathways. TGF-β is known for its activation of the TAK1 (TGF-β activated kinase 1) MAPK. TAK1 can then activate by phosphorylation the p38 (on the Thr180/182) and/or JNK (on the Thr183/Tyr185) pathways^17–19^. The activation of TAK1 is via the TRAF6 protein. Following TGF-β activation, TRAF6 is polyubiquitynated and this step leads to TAK1 activation. This activation is dependent of the binding between TRAF6 and the TGF-β receptor complex^20^. In another study, it was shown that TRAF6 ubiquitination leads to the subsequent ubiquitination of TAK1, which is TGF-β ligand dependent. This modification is necessary for TAK1 phosphorylation and kinase activity^20–22^. TRAF6 was also shown to be important for EMT, which confirms the importance of TGF-β non-canonical pathway in the process^20^.

During axolotl limb regeneration, the TGF-β canonical signaling pathway is essential. If the pathway is blocked with SB-431542, the SMADs are not phosphorylated and formation of the blastema does not occur^1,2,23^. It was shown previously that TGF-β is present in axolotl skin, thus stored in the ECM and ready to be activated following an injury^24^. This explains in part the fast response and the presence of active TGF-β right after the amputation. In other regenerating models, TGF-β has also been shown to be important. The wound epidermis formation is blocked in Xenopus tail regeneration when the animals are treated with 100 μM of SB-431542^25^. TGF-β has also been shown to be necessary for heart regeneration in zebrafish^26^.

As for the non-canonical pathways in regeneration, JNK has been shown to be necessary for blastema formation in the planarian model. Treating planarian with the JNK inhibitor SP-600125 prevents proliferation and blastema formation. In the same study, they show that wound healing is also inhibited by SP-600125^27^. In zebrafish wound closure of a partial/full thickness wound is driven by the JNK pathway^28^. The MAPK p38, another non-canonical TGF-β target, was shown to regulate heart regeneration in zebrafish^29^. In the planarian, p38 inhibition with SB-203580, a p38 specific inhibitor, did not show any effect on blastema formation^26^. Taken together, all these studies show that both canonical and non-canonical TGF-β pathways are involved regeneration in different animal models.

In the present study, we have performed experiments showing that different EMT markers are expressed during the process of limb regeneration. We also show that EMT is regulated in a biphasic manner during regeneration. The expression of the different markers correlates with the wound closure phase (very early in the process in which keratinocytes migrate) and redevelopment phase (the latter stage of regeneration). However, not all genes are showing this bi-phasic regulation in both phases. These results point to two different EMT signatures during regeneration. To understand the importance of signaling regulating the expression of theses EMT markers during the first phase (wound healing/closure), we used a pharmacological approach. We used inhibitors targeting the SMADs, p38 and JNK. We show the specificity of all the inhibitors, as each of them do not block the other’s activation. Inhibiting SMADs, p38 and JNK individually showed no effect on wound closure and EMT markers expression. To observe an effect on EMT, animals had to be treated with a combination of inhibitors blocking SMAD, p38 and JNK. Consequently, wound closure was significantly delayed. The results demonstrate that all three pathways participate in the wound closure process. To further confirm that JNK and p38 roles in EMTs and wound closure are TGF-β dependent, we took advantage of the TAK1 inhibitor and showed similar results from the ones we observed when using the specific inhibitors to both proteins. All together, these results support the hypothesis that EMTs are important during limb regeneration. Also, their regulation is dependent in part on the TGF-β canonical and non-canonical pathways.

## Results

### EMT markers are regulated during limb regeneration

**Figure 1:**
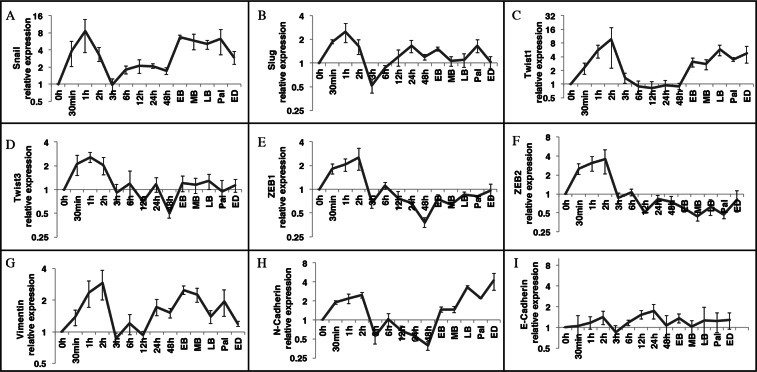
RT-qPCR on EMT markers during axolotl limb regeneration. Tissue harvested at different time points during regeneration. Each time point is relativized on the T = 0 h (basal level). (**A**–**H**) Relative expression of different mesenchymal markers; (**A**) Snail, (**B**) Slug, (**C**) Twist1, (**D**) Twist3, (**E**) ZEB1, (**F**) ZEB2, (**G**) Vimentin, (**H**) N-Cadherin. (**I**) Relative expression of epithelial marker E-Cadherin. Normalized using GAPDH. Mean ± s.e.m, N = 3.

The regulation of known EMT markers was evaluated during the regeneration process. We started by measuring the expression of EMT markers by RT-qPCR on a regeneration time course covering all the major stages of regeneration. Many genes are known to be EMT markers. In this study, we looked at the transcription factors Snail, Slug, Twist1, Twist3, ZEB1 and ZEB2. The expression of mesenchymal markers Vimentin and N-Cadherin was also assessed as was the epithelial cell marker E-Cadherin. We performed RT-qPCR on a full regeneration time course and normalized each time point to the t = 0 h (considered basal level). The results show an up-regulation of Snail, Slug, Vimentin, N-Cadherin, ZEB1, ZEB2, Twist1 and Twist3 from 30 minutes to 3 h hour post-amputation (Fig. 1A–H). Interestingly, expression of these mesenchymal markers correlates with the wound closure process following injury. These RT-qPCR results also show a second phase of expression of EMT markers. When we look at the re-development phase later in regeneration, we observe that Snail, Twist 1, Vimentin and N-Cadherin are up-regulated (Fig. 1A,C,G,H). This shows a bi-phasic regulation of EMT with a specific gene expression signature for each phase.

Seeing how EMT markers specific to the mesenchymal state were regulated, we looked at the epithelial marker E-Cadherin, known to be down-regulated in EMT. Results show that E-Cadherin is maintained to a relatively basal level throughout the regeneration process (Fig. 1I). By comparing these different results, we observe that E-Cadherin is at a very low level compared to mesenchymal markers such as Snail and Vimentin during regeneration.

Altogether, these results suggest that EMT markers are expressed during regeneration and expression of various markers display different signatures depending if we look at the wound closure or the redevelopment phase.

### EMT markers expression is localized in migrating keratinocytes and in blastema

**Figure 2:**
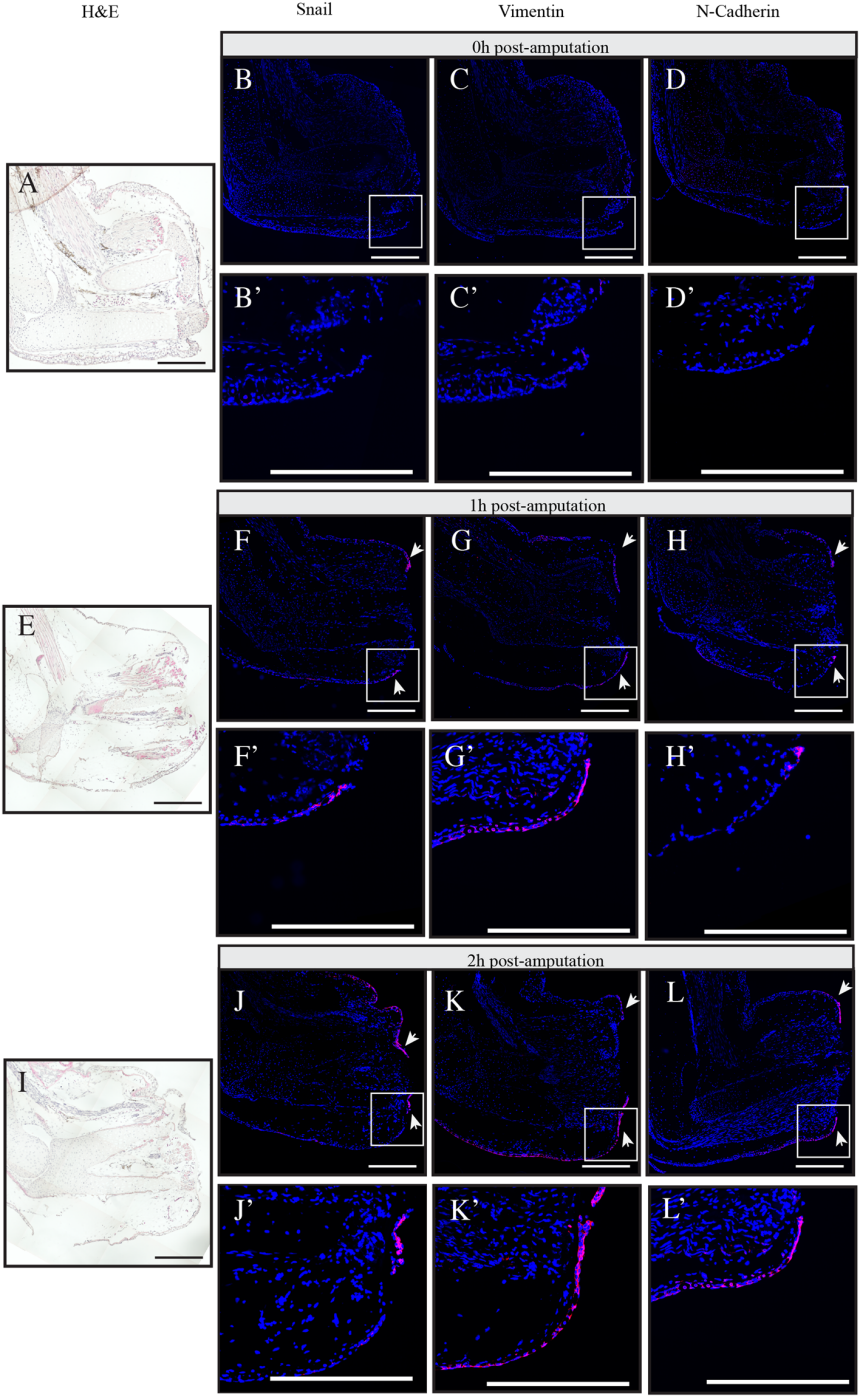
*In situ* hybridization using tyramide signal amplification showing the expression of EMT markers during regeneration. Different regeneration time points (**A**–**D**) Time 0 h, (**E**,**F**) Time 1 h post-amputation, (**I**–**L**) Time 2 h post-amputation. (**A**,**E**,**I**) Hematoxylin and eosin (**H** & **E**) coloration. Overlay of nuclei staining with DAPI (blue) and *In situ* hybridization with Cy5 (red) for (**B**-**B**’,**F**-**F**’,**J**-**J**’) Snail, (**C**-**C**’,**G**-**G**’,**K**-**K**’) Vimentin, (**D**-**D**’,**H**-**H**’,**L**-**L**’) N-Cadherin. White boxes represent magnified areas. White arrows show the signal in migrating epithelia. Scale bars are 200 μm. Composite images are shown.

The previous section showed a regulation of EMT markers during regeneration. RT-qPCR offers good quantitative results but it does not provide information on the localization or cells expressing the genes of interest. *In situ* hybridization combined with Tyramide Signal Amplification (TSA) was used to determine which cells were expressing the markers studied in Fig. 1. We were able to design working probes specific for Snail, Vimentin and N-Cadherin (Fig. 2). Interestingly, the three genes were not detected in t = 0 h, as seen in the RT-qPCR results in Fig. 1. The expression is observed in migrating keratinocytes 1 h and 2 h post-amputation. Following wound closure at 3 h post-amputation, we still observed expression of these markers in the AEC. The expression is reduced to an undetectable level at 48 h post-amputation (Supplementary Fig. 1).

The RT-qPCR results in Fig. 1 also showed expression of some of the markers during redevelopment phase. We performed *in situ* hybridization for later time points to compare the different cell types expressing EMT markers at the early stage of regeneration vs later stages. Starting at early bud, we observe expression of these markers in blastemal mesenchymal cells (Supplementary Fig. 1).

We also looked at the epithelial marker E-Cadherin (Supplementary Fig. 2) and we see no expression in migrating epithelial cells. We start seeing E-Cadherin expression when the keratinocytes just finished migrating over the open wound to close it.

Together these results corroborate with the RT-qPCR results (Fig. 1).

### JNK, p38 and TGF-β pathways as potential regulators for EMT markers

**Figure 3:**
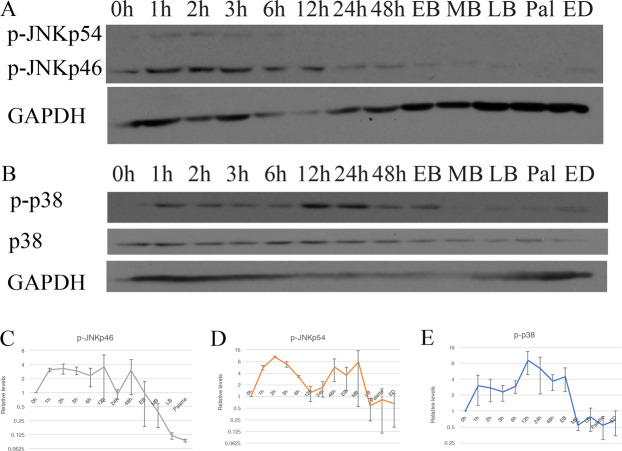
JNK and p38 activation during axolotl limb regeneration. Western Blot analysis showing (**A**) p-JNK and (**B**) p-p38 and total p38 during the course of normal limb regeneration. GAPDH is used as a loading control. (**C**–**E**) Band quantification normalized on GAPDH and relativized on T = 0 h for (**C**) p-JNKp46, (**D**) p-JNKp54 and (**E**) p-p38. Means ± s.e.m. N = 3.

Seeing how EMT markers were regulated during regeneration, we wanted to understand what signaling pathways were responsible for modulating their expression. TGF-β is a well-known pathway for regulating EMT. We have previously shown that SB-431542, a TGF-β/SMAD canonical pathway inhibitor prevents blastema formation at 25 µM and blocks phosphorylation of Smad2 and Smad3^1,2^. With SB-431542 (inhibitor of the canonical pathway) treatments, no effect was observed on wound closure^2^. TGF-β also acts via non-canonical pathways and can activate MAPKs such as p38 and JNK. We wondered whether these pathways could play a role in wound closure. In addition, p38 and JNK have been shown to be involved in modulating the EMT process. We looked at both p38 and JNK pathways to assess whether they could be involved in the EMT process during regeneration. Since it was never done before, we first looked at the regulation of both proteins during the regeneration process (Fig. 3). For JNK, we used an antibody targeting the phosphorylation sites at Thr183 and Tyr185. The antibody can detect the two isoforms of JNK known as p46 and p54. Both isoforms are quickly activated, starting around 1 h post-amputation. The JNKp54 is down regulated at 6 h. The p46 isoform’s activation is maintained up to EB (Fig. 3A). For p38, we used an antibody recognizing the Thr180/Tyr182 phosphorylated site. We observed phosphorylation very early in the process, starting at 1 h post-amputation, up to EB (Fig. 3B). The expression patterns of the two phosphorylated proteins correspond to the expression of the EMT markers during wound closure, as they are active in the first few hours following amputation. These results suggest that p38 and JNK are potential regulators of EMT at the beginning of regeneration since their activation corresponds to the upregulation of EMT markers during regeneration.

### Effect of SB-431542, SB-203580 and SP-600125 on phosphorylation and wound closure

**Figure 4:**
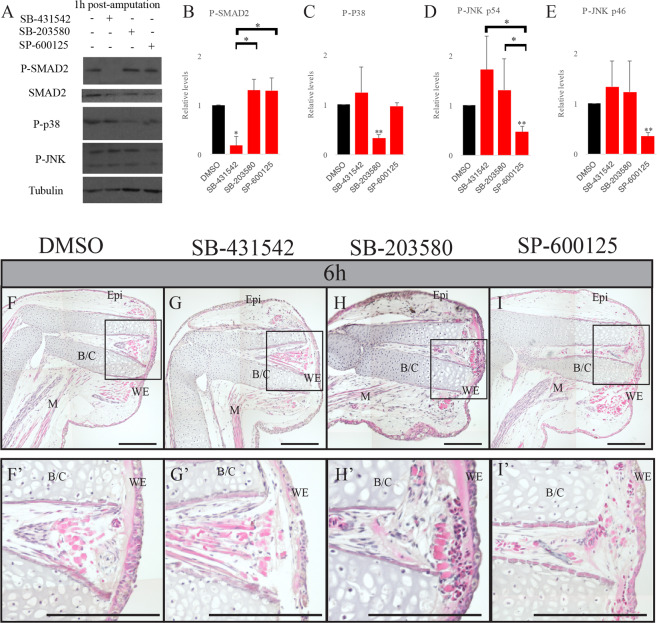
SB-431542, SB-203580 and SP-600125 treatments show no effect on wound closure. (**A**) Western Blot analysis showing the effect of SB-431542, SB-203580 and SP-600125 on SMAD2, p38 and JNK phosphorylation 1 h post-amputation. Tubulin is used as a loading. (**B**–**E**) Band quantification of p-SMAD2, p-p38 and p-JNK and normalization on Tubulin (n = 3). A one sample Student’s t test was performed to compare each treatment to the DMSO control (relative value adjusted to 1 to allow comparison between experiments), represented by the asterisks over each bar in the graph. A one-way ANOVA was done to compare the different treatments together, represented by the asterisks over the brackets. *p < 0.05, **p < 0.005, Means ± s.e.m. Hematoxylin and eosin staining on treated axolotl limbs 6 h post-amputation. (**F**,**F**’) DMSO treated animals (**G**,**G**’) SB-431542 treated animals (**H**,**H**’) SB-203580 treated animals (**I**,**I**’) SP-600125 treated animals. All treatments show no effect on wound closure as the wound epidermis is formed as in the control. Scale bars are 200 μM. Composite images are shown. (WE) wound epidermis, (M) Muscle, (Epi) Epidermis, (B/C) Bone and cartilage. The black rectangles show the fully formed wound epidermis in the magnified panels (**F**’**,G**’**,H**’**,I**’).

A pharmacological approach was used to understand if these different signaling pathways are important for wound closure and EMT during regeneration. SB-431542 was used to block the canonical TGF-β pathway. We showed in a previous study that SB-431542 blocks p-SMAD2 and p-SMAD3^1^. SB-203580 was used to block p38 and SP-600125 to block JNK. Since the 2 latter inhibitors have never been used in axolotls before, we tested these inhibitors to make sure that they were functional and specific in regenerating axolotl limbs. The 1 h post-amputation time point was selected since it corresponds to the peak of EMT marker expression. In Fig. 4, we show that SB-431542 blocks p-SMAD2 without reducing the levels of p-JNK and p-p38 (Fig. 4B). Similar results are observed while treating with SB-203580 as p-p38 is reduced but not p-SMAD2 or p-JNK (Fig. 4C). When animals are treated with SP-600125, p-JNK is reduced but not p-SMAD2 and p-p38 (Fig. 4D,E). The effects observed for p-SMAD2 when treated with SB-431542, p-p38 when treated with SB-203580, p-JNKp46 and p-JNKp54 when treated with SP-600125 where all significantly inhibited compared to the DMSO control. The levels of all the other conditions were not affected by the treatments. One-way ANOVA tests allowed us to compare the different treatments to each other. When looking at p-SMAD2 we observe a significant effect when treated with SB-431542 vs treatment with SP-600125 or SB-2013580. When looking at p-JNKp54, treatment with SP-600125 shows a significant effect compared to SB-431542 or SB-2013580. The second isoform, p-JNKp46 only shows a significance with a p < 0.1, but a clear tendency is shown that there is a difference between the SP-600125 and the 2 other treatments. The same is observed for p-p38 where the tendency shows that p-p38 is specifically reduced by the SB-203580 compared to the two other treatments.

These results confirm that we are able to block TGF-β canonical and non-canonical pathways with pharmacological inhibitors in regenerating limbs.

To understand whether p38 and JNK had a role in wound closure, we treated animals with both inhibitors and did histology to assess epidermal migration. As Fig. 4 shows, at 6 h post-amputation we observe a fully closed wound in all treatments (Fig. 4F–I). Treatment with SB-431542 also causes no effect on wound closure confirming the results from Levesque *et al*.^2^ (Fig. 4G). Seeing how none of these had any effects on wound closure, we wondered if by blocking one of the proteins the others could take over and drive wound closure.

### Effect of combination of inhibitors on wound closure

**Figure 5:**
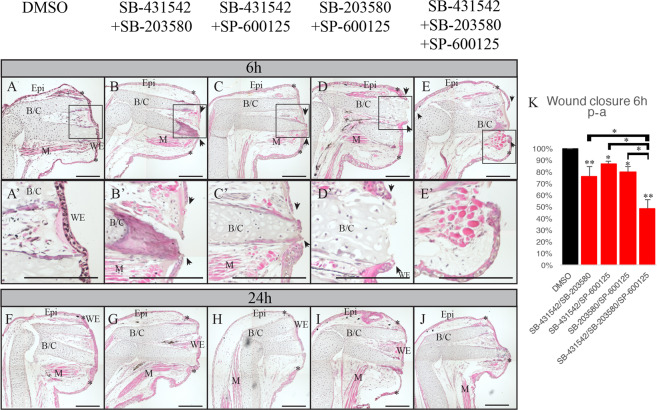
Effect of combinations of SB-431542, SB-230580 and SP-600125 on wound closure. Hematoxylin and eosin staining on treated axolotl limbs. (**A**–**E**) 6 h post-amputation (**A**) DMSO control, the wound is fully closed; (**B**) SB-431542 and SB-203580 treatment, wound epidermis is not formed; (**C**) SB-431542 and SP-600125 treatment, wound epidermis is not formed; (**D**) SB-203580 and SP-600125 treatment, wound epidermis is not formed; (**E**) SB-431542, SB203580 and SP-600125 treatment, wound epidermis is not formed. (**F**–**J**) 24 h post-amputation, (**F**) DMSO control, the wound is fully closed; (**G**) SB-431542 and SB-203580 treatment, the wound is fully closed; (**H**) SB-431542 and SP-600125 treatment, the wound is fully closed; (**I**) SB-203580 and SP-600125 treatment, the wound is fully closed; (**J**) SB-431542, SB203580 and SP-600125 treatment, the wound is fully closed. (**K**) Average percentage of wound closure of each treatment 6 h post-amputation. A Student’s t test was performed to compare each treatment to DMSO control (fully closed wound, 100%), represented by the asterisks over each bar in the graph. A one-way ANOVA was done to compare the different treatments together, represented by the asterisks over the brackets. *p < 0.05, **p < 0.005, Means ± s.e.m. Scale bars are 200 μM. Composite images are shown. (WE) wound epidermis, (M) Muscle, (Epi) Epidermis, (B/C) Bone and cartilage. The black rectangles show the fully formed wound epidermis or the open wounds, which is magnified in (**A**’,**B**’,**C**’,**D**’,**E**’). Asterisks show the site of amputation. Black arrowheads show the migrating keratinocytes.

To observe if the different pathways could take over one another, we tested different combinations of treatments with the different inhibitors on animals. Wound closure was assessed in animals treated with different combinations of treatments using histology and compared them to DMSO control 6 h post-amputation (Fig. 5A). In a normal limb of animals 4–5 cm long, the wound is closed between 2–3 h post-amputation (Supplementary Fig. 3). Therefore, 6 h was a good time-point to observe a possible effect on wound closure. Following the treatments with combinations of SB-431542/SB-203580, SB-431542/SP-600125 or SB-203580/SP-600125, we observe that the wound is not fully closed which indicates a delay compared to the DMSO control (Fig. 5A vs B–D).

By treating with all three inhibitors, we see the wound is not healed after 6 h indicating that a significant inhibition is occurring (Fig. 5E).

We also show results at 24 h post-amputation. At this time point, all of the treated animals show a closed wound (Fig. 5F–J). This result shows a delay in the process but not a full inhibition. In panel K of Fig. 5 we determined the percentage of closure for each treatment and observed a more severe inhibition when all drugs were combined suggesting some interaction between canonical and non-canonical signaling.

We also wanted to look at the effect of these treatments on the expression of EMT markers. We again used the 1 h post-amputation time point as it showed the maximum expression for all markers. When doing RT-qPCR on the combination of only 2 inhibitors, very few to no inhibition is observed. When we combine of all three inhibitors (blocking all three pathways), we see an effect on the expression of most EMT markers. We observe a significant inhibition on Slug, ZEB2, Twist1, Twist3, Vimentin and N-Cadherin (Supplementary Fig. 4).

### Effect of TAK1 inhibition on wound closure and p38/JNK phosphorylation

**Figure 6:**
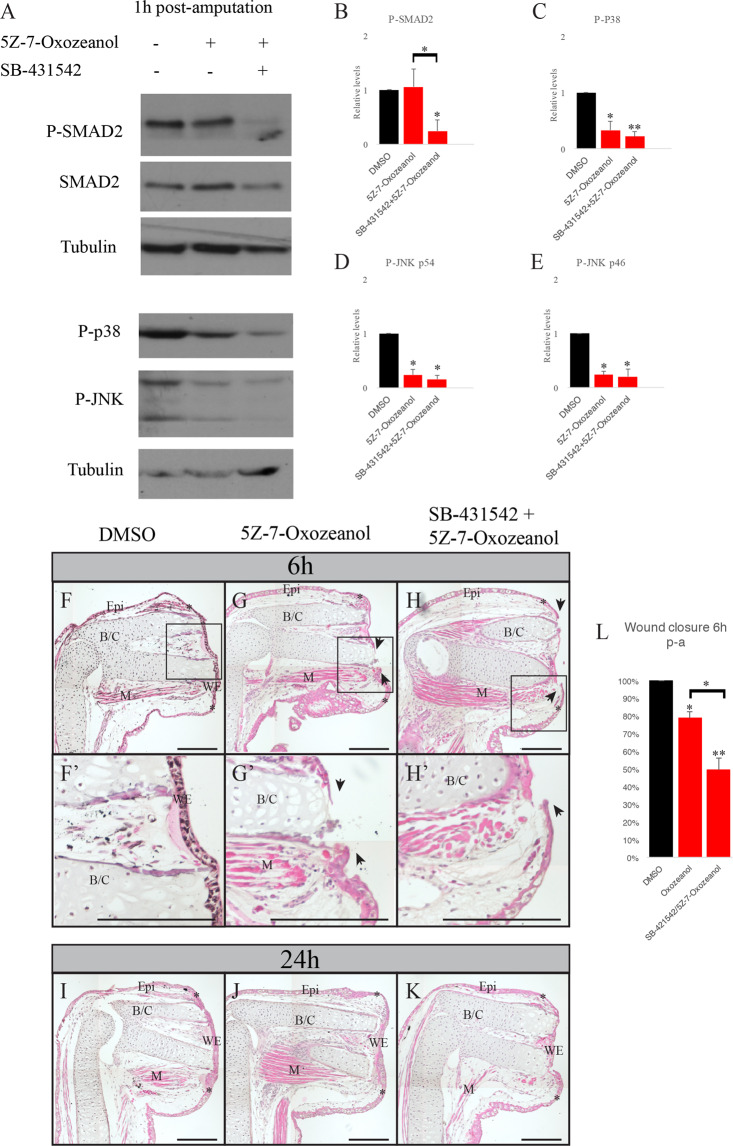
Effect of 5Z-7-Oxozeanol on protein phosphorylation and wound closure. (**A**) Western Blot analysis showing the effect of 5Z-7-Oxozeanol and SB-431542 on SMAD2, p38 and JNK phosphorylation 1 h post-amputation. Tubulin is used as a loading. (**B**–**E**) Band quantification of (**B**) p-SMAD2, (**C**) p-p38 and (**D**,**E**) p-JNK, normalization on Tubulin (N = 3). A one sample Student’s t test was performed to compare each treatment to DMSO control (relative value of 1 to compare between experiments), represented by the asterisks over each bar in the graph. A one-way ANOVA was done to compare the different treatments together, represented by the asterisks over the brackets. *p < 0.05, **p < 0.005, Means ± s.e.m. (**F**–**K**) Hematoxylin and eosin staining on treated axolotl limbs. (**F**–**H**) 6 h post-amputation (**F**,**F**’) DMSO control, the wound is fully closed (**G**,**G**’) 5Z-7-Oxozeanol treatment, wound epidermis is not formed (**H**,**H**’) Combination of 5Z-7-Oxozeanol and SB-431542 treatment, wound epidermis is not formed. (**I**–**K**) 24 h post-amputation. (**I**) DMSO control, the wound is closed (**J**) 5Z-7-Oxozeanol treatment, the wound is closed (**K**) Combination of 5Z-7-Oxozeanol and SB-431542 treatment, the wound is closed. (**L**) Average percentage of wound closure of each treatment 6 h post-amputation. A one sample Student’s t test was performed to compare each treatment to DMSO control (fully closed wound, 100%), represented by the asterisks over each bar in the graph. A one-way ANOVA was done to compare the different treatments together, represented by the asterisks over the brackets *p < 0.05, **p < 0.005, Means ± s.e.m. Scale bars are 200 μM. Composite images are shown. N = 3. (WE) wound epidermis, (M) Muscle, (Epi) Epidermis, (B/C) Bone and cartilage. The black rectangles show the fully formed wound epidermis or the open wound, which is magnified in (A’,B’,C’,D’,E’). Asterisks show the site of amputation. Black arrowheads show the migrating keratinocytes.

The proteins p38 and JNK can be activated via many different signaling pathways, not only TGF-β^30^. Their activation by TGF-β is known to go through the MAPK TAK1 (TGF-β activated kinase 1)^17,30,31^. To confirm that the involvements of p38 and JNK in limb regeneration EMTs are TGF-β dependent, we took advantage of the commercially available TAK1 inhibitor, the 5Z-7-oxozeanol. It was shown to block TAK1 activity and p38/JNK phosphorylation^32^. First, we had to assess if the inhibitor was specifically blocking the TGF-β non-canonical pathway, but had no effect on the canonical pathway. In Fig. 6, we see that p-p38 and p-JNK are reduced by a treatment with 5Z-7-Oxozeanol. Inhibition levels are very similar between the 5Z-7-Oxozeanol and the p38 and JNK specific inhibitors. All the proteins observed showed a 70% inhibition except for the p-JNKp54 isoform that showed a 60% inhibition when treated with the SP-600125. Also, p-SMAD2 is not affected when we only treat the animals with the TAK1 inhibitor, but is when we add the SB-431542 (Fig. 6A,B).

As we did in the previous section, we looked at wound closure following the treatments with the TAK1 inhibitor. Treating only with the 5Z-7-Oxozeanol blocks p38 and JNK and shows an delay in wound closure 6 h post-amputation. The migrating keratinocytes have not closed the wound. When we combined SB-431542 and 5Z-7-Oxozeanol, we observed that the wound is not closed 6 h hour post-amputation, and the effect seems stronger as the wound has not begun to heal (Fig. 6F–H,L). At 24 h post-amputation, the wound is fully closed confirming a delay, but again not a full inhibition of the process (Fig. 6I–K). These results indicate that the regulation of p38 and JNK during EMT and wound closure are TGF-β dependent.

We looked at the effect of these treatments on the expression of the EMT markers. When only treating with the 5Z-7-Oxozeanol we observe limited inhibition on EMT markers as only ZEB2 and Slug are reduced by the treatment. When blocking both canonical and non-canonical pathways with the combination of SB-431542/5Z-7-Oxozeanol we observe a stronger inhibition. The genes Slug, ZEB2, Twist3, Vimentin and N-Cadherin are all significantly reduced while Snail, ZEB1 and Twist1 don’t show a significant inhibition (Supplementary Fig. 5).

## Discussion

Epithelial to mesenchymal transitions are present in many different biological processes. From development to cancer progression and wound healing, these behavioral changes in epithelial cells are important for cellular migration. During EMT, cells lose their polarity and anchorage to gain the ability to migrate^12^. In the case of limb regeneration almost no data is available regarding EMT and how it is controlled. Regeneration is a combination of wound healing followed by a proliferation phase and a development phase. Seeing how EMTs are involved in these cellular processes in other systems, it was interesting to study their expression during regeneration.

The expression profile experiments demonstrated that there was a wound healing and a redevelopment stage of EMT during limb regeneration. Wound epidermis formation occurs very early following amputation and is a crucial step for regeneration to proceed. Both the wound healing and redevelopment phases are of interest but it was decided to focus on the wound healing for the present study as it coincides with TGF-β activation as our lab has shown in previous publications^1,2^.

RT-qPCR and *in situ* hybridization results not only showed a regulation of multiple EMT markers, but also that two EMT signatures can be observed between the wound healing and redevelopment phases of regeneration. All the mesenchymal markers we looked at were up-regulated during the wound healing phase. As for E-Cadherin, it did not show much regulation in qPCR results during the whole regeneration process. When looking at the redevelopment phase, only Snail, Twist1, N-Cadherin and Vimentin were increased. During the wound closure phase, the *in-situ* hybridization shows that the EMT genes are localized in the migrating keratinocytes. The genes observed in redevelopment phase were localized in the blastema which is made of mesenchymal cells. E-Cadherin was not observed in migrating keratinocyte, but its expression was detected when the wound closes and the epithelial cells anchor themselves together.

The RT-qPCR and *in situ* hybridization results corroborated each other and allowed the identification of two EMT phases. In the first few hours following amputation, expression of EMT markers is observed in migrating keratinocytes. This expression is short lived, and by 3 h post-amputation it is fading in keratinocytes. At 48 hours, we did not detect signal anymore. Later, as shown with RT-qPCR results, expression is detected at time points when the blastema starts to form at the early bud stage. The signal grows stronger as the redevelopment phase progresses up to early differentiation stage.

In this study, we did not focus on the redevelopment phase, but in future studies it would be of interest to evaluate if EMT is important at this point during regeneration. Other studies have shown that EMT genes are important during limb bud development. Seeing how the redevelopment phase of regeneration has some similarity with embryonic development, these EMT might play a role in regeneration similar to what is observed during embryonic development. The Twist transcription factors have been shown to play an important role in the growth and differentiation of the mouse limb bud^33^. Interestingly, this potential importance of Twist during limb regeneration has also been observed^34^. Another study looked at the Slug transcription factor during chick limb development. This paper showed that Slug was expressed in undifferentiated mesenchyme and was important for the maintenance of the progress zone^35^. It is possible that these EMT genes during limb redevelopment are there to maintain the mesenchymal cells of the blastema into a dedifferentiated state, as it is observed in development.

The wound closure steps result in the formation of the wound epidermis. If the epidermis is mechanically removed the limb will never regenerate^36^. An interesting aspect of wound closure in salamander regeneration is the speed with which it occurs. In mammals, reepithelization can take many days or even weeks to complete^37^. Wound closure is usually complete within a few hours in axolotls^37,38^. EMTs have been shown to play an important role in wound healing in mammals in two ways. Some evidence shows that myofibroblasts can arise from the epidermal cells that underwent an EMT program^39^. In the case of axolotl, myofibroblasts are not detectable in axolotl^37,40^. The second case of EMT in wound healing is for the migration of keratinocytes during reepithelization. In the case of reepithelization, a lot of EMT traits have been shown, like cell motility and the reduction of epithelial traits^41^. At the molecular level, some EMT characteristics have been observed. Vimentin knockout mice show a major defect in the reepithelization process, but also in their EMT program in the case of an excision injury^42^. Slug knock-out mice show a reduced reepithelization^43,44^. Slug is the only EMT transcription factor that has been shown to have a functional role in reepithelization. In the axolotl, we observe an up-regulation of 6 transcription factors (Fig. 1)^41^. We also observe a correlation in the delay of wound closure with the reduced expression of Slug, Twist1, Twist3 and ZEB2 suggesting they play a part in the wound closure process (Supplementary Figs 4 and 5). It could be that the faster rate of wound epidermis formation in axolotl compared to mammal results from higher number of EMT inducing genes involved in the reepithelization process.

TGF-β/SMAD canonical pathway had no effect on wound closure rates in axolotls^2^. Our Western Blot results showed that the non–canonical factors p38 and JNK were active during this wound closure phase. Considering these results, we also tested their importance in wound closure. Treating these animals with specific inhibitors for JNK and p38 showed no effect either on wound closure. To ascertain the specificity of the pharmacological inhibitors we looked at the phosphorylation of the different targets. We showed that each inhibitor blocks its predicted target, but also that they were specific and did not inhibit the other targets. Seeing how complex limb regeneration is, we thought that when blocking one pathway another one could take over or compensate and complete the wound closure, since the Western Blot results showed no inhibition of the non-targeted proteins.

Treating animals with combinations of SB-431542/SB-203580, SB-431542/SP-600125 and SB-203580/SP-600125 showed a reduced rate in the wound closure at 6 h post-amputation with all of these treatments not fully closed. When treating with all three inhibitors at the same time, we observed an even more important delay in wound closure. In this case the leading keratinocytes don’t seem to have started to migrate at 6 hours post-amputation as they are still very close to the amputation site. Thus, when blocking all 3 pathways, we get a stronger effect on wound closure. These results could mean two things. First, either the participation of all three pathways are necessary for the process. Secondly, they could compensate for each other during wound healing, if one is blocked the two others can take over.

JNK and p38 are proteins central to many signaling pathways. Consequently, we could not determine if their activation was TGF-β dependent using their specific inhibitors. The 5Z-7-Oxozeanol blocks the kinase action of TAK1, the kinase responsible for TGF-β dependent activation p38 and JNK. With both treatment combinations of SB-203580/SP-600125 or 5Z-7-Oxozeanol, we start to understand how the signaling occurs during regeneration. The effect of 5Z-7-Oxozeanol on wound closure was similar to the combination of SB-203580/SP-600125 leading to the conclusion that activation of p38 and JNK during wound closure was TGF-β dependent. The combined treatment with SB-431542 and 5Z-7-Oxozeanol showed similar results when compared to the combined treatment with SB-431542/SB-203580/SP-600125. We saw a stronger effect on wound closure than when only treating with 5Z-7-Oxozeanol. Following these results, we also did the RT-qPCR of the different treatments on EMT genes. We see the highest level of inhibition with both SB-431542/5Z-7-Oxozeanol and SB-431542/SB-203580/SP-600125 treatments. This correlates to the histology results as we observe a more pronounced effect on wound closure in these treatments.

Results show that we can only slow down the process of wound closure as when we look at 24 h post-amputation with a treatment of SB-431542/SB-203580/SP-600125 or with the SB-431542/5Z-7-Oxozeanol the wound is closed. Seeing how we can block all these pathways, we believed we would be able to block wound closure. As mentioned earlier, the axolotl limb is a complex structure with many cell types. In addition, multiple pathways beside TGF-β could be involved in wound closure. Not all EMT genes were reduced following TGF-β inhibition (e.g. Snail, ZEB1 and Twist1) which would support/suggest the presence of other pathways being active. The participation of another pathway would make sense considering the importance of EMT processes in regeneration and wound healing.

In this paper, we show that EMT is important in the context of regeneration since the process is tightly linked with keratinocytes migration and wound closure. This is the first step for perfect healing, since it leads to the formation of the apical epidermal cap (AEC), a fundamental structure for regeneration^36^. We also show how robust wound closure is in axolotls. Even if we block both canonical and non-canonical TGF-β pathways the animal is still capable of eventually closing its wound, showing the importance of the process. The study also provides additional data on EMTs in wound healing in an *in vivo* model. Future experiments looking at alternate pathways involved in mediating EMTs will be important to fully understand this process during regeneration.

## Material and Methods

### Animal care

Axolotl were purchased from Ambystoma Genetic Stock Center (Lexington, KY) and caring was done according to Denis *et al*.^1^. For all experiments, animals were between 4 and 5 cm long, and were about 4 months old. Each drug was added to the water at the determined concentration. The SB-431542 was purchased from Sigma-Aldrich (S4317), the SB-203580 and SP-600125 from Selleckchem (S1076 and S1460). All inhibitors were diluted at a concentration of 10 mM in DMSO (Fisher Scientific BP231-100). The appropriate amounts of 10 mM stock were then diluted to the desired concentration for each experiment directly in 5 mL of 20% Holtfreter’s solution for each animal. The solutions were changed daily and all treatments were started 24 hours before amputation. For details of each inhibitor see Table 1. All animal experimentations were approved by the animal care and ethics committee of Université de Montréal (approval code: CDEA#17-123) which is recognised by the Canadian Council for Animal Care.

**Table 1:** Details of the inhibitors used.

Inhibitor	Company	Doses used
SB-431542	Sigma Aldrich (S4317)	25 µM
SB-203580	Selleckchem (S1076)	50 µM
SP-600125	Selleckchem (S1450)	5 µM
5Z-7-Oxozeanol	Millipore Sigma (499610)	1 µM

### RNA isolation and RT-qPCR

Regenerating limb tissues were harvested including 1 mm of stump from the amputation level for each time-point studied. Tissues were shredded in Invitrogen™TRIzol™Reagent (Thermo Fisher Scientific, 15596026) using syringes (18–23 gauges). RNA was isolated following manufacturers guideline. The RNA quantification was done according to Denis *et al*.^1^. For each sample, 200 ng of RNA were reversed transcribed (RT) using SuperScript® VILO™ cDNA Synthesis Kit (Thermo Fisher Scientific, 11754050), using the manufacturer’s guidelines. RTs were diluted 1:8 in RNAse free water to a final volume of 160 µL.

For qPCR, 2 µL of cDNA were used per reaction in our lab made mastermix [(1X Standard *Taq* reaction buffer (New England Biolabs), 0.5 mM MgSO4, 0.33X SYBR Green I (Life Technologies, S7580), 0.2 mM dNTP mix (Thermo Fisher Scientific, 10297018), 0.5 units of Hot Start *Taq* DNA polymerase (New England Biolabs, M0495S)] and the volume was completed to 20 µL with RNAse free water. Primers (forward and reverse) for each gene of interest were added at a final concentration of 0.25 μM. qPCR reactions were done on the LightCycler® 480 Instrument (Roche). The program used was: 2 minutes denaturation at 94 °C, 40 cycles of [15 sec. 94 °C denaturation, 30 sec. 60 °C annealing, 60 sec. 72 °C elongation]. A 10 minutes melt curve was done to confirm primer specificity. Primers are listed in Table 2.

**Table 2:** List and sequence of the RT-qPCR primers.

Gene	Forward sequence	Reverse sequence
GAPDH	TTGTCCTACGTGTGCTGTCTGT	TCACACAGTGCCAAGATAAGTGTT
Snail	AGAAGGAGTATGTCAGCCTGGG	TGAGAACGGCTTTTCACCTGT
Slug	ATGCTGTAGGGAGAGCGTGGAG	CAGCTCGCTGTAGTTGGGCTTCT
Twist1	ACATTGCCCACACTCCATTT	TAGACACCGGATCCATCAGC
Twist3	GCTATGCCTTCTCGGTTTGG	ACTTTCAGGTGGGGGTTCCT
ZEB1	GAGGAGGACGAGAAAGATGTTGA	CACTCATGGGTATGTCCTCTTCC
ZEB2	TGTTTATGGGAAGTTAGGTCAGCA	GTCCATCGAATGATCTCCAT
Vimentin	GCCTTCAGGACGAAATCCAG	CAGTGATCCTGCACTCCTCG
N-Cadherin	CAGCCGGATACTCTGGAACC	GTCCCCAATGTCACCAGGAT
E-Cadherin	CTTTGTTGCCCCCTGATGA	TACGCATTACGTCTGGTCGG

### *In situ* hybridization using Tyramide Signal Amplification (TSA)

Regenerating limbs were harvested at desired time points post-amputation and fixed overnight at 4 °C in 4% PFA in 0.7X PBS. Samples were paraffin embedded, cut to a thickness of 10 μm using a microtome, dried overnight, deparaffinized in toluene and rehydrated in decreasing concentration of ethanol. Following rehydration, samples were treated with proteinase K (Thermo Fisher Scientific, 25530015) at 5 μg/mL in 0.1 M TrisHCL pH7.5, 50 mM EDTA pH8.0 in PBS-T. Samples were then fixed 5 minutes in 4% PFA in 0.7X PBS and rinsed in 0.7X PBS. Pre-hybridization was done at 42 °C for 1 hour in hybridization solution (50% Formamide (Fisher Scientific, BP227-100), 2X SSC, 1X Denhardt’s, 0.5% Sodium pyrophosphate (Sigma Aldrich, P8135), 0.5% SDS, 25 μg/mL salmon sperm DNA (Thermo Fisher Scientific, 15632011), 250 μg/mL yeast tRNA, 0.25 M TrisHCl). For hybridization, the probes (generated using the primers in Table 3) were diluted at 5 μg/mL in hybridization solution. Hybridization was done O/N at 42 °C. On the next day, slides were rinsed successively in 2X SSC, 2XSSC/50% formamide and 1X SSC at hybridization temperature, and then rinsed in 0.1X SSC and 0.7X PBS at room temperature. Samples were blocked 1 h at room temperature in 10% sheep serum (Sigma Aldrich, S3772) in MAB-T (100 mM NaCl, 100 mM maleic anhydride, 0.3% Triton, pH7.5). Incubation with anti-digoxigenin (Roche, 11093274910) 0.002 μg/µL in blocking solution O/N at 4 °C. The next day, slides were rinsed 3 times in MAB-T and incubated with anti-mouse antibody (Biorad, 170–6516) 1:400 in blocking solution for 1 hour at RT. Samples were rinsed 3 times in MAB-T and incubated 7 minutes with Tyramide-CF640R (Biotium, 92175) in 0.005% H_2_O_2_ 0.7X PBS and rinsed 3 times in 0.7X PBS. Mounting was done using ProLong® Gold antifade reagent with DAPI (Thermo Fisher Scientific, 36931). Slides were analysed with a Zeiss Axio Imager M2 Optical Microscope (Zeiss, Munich, Germany). Pictures were taken with the Axiocam Monochrom Mr using tiling. A composite image was made followed by a stitching step and cropped to show the area of interest.

**Table 3:** Sequence of primers used for probe synthesis.

Gene	Forward sequence	Reverse sequence (with T7 promoter sequence)
Snail	AGAAGGAGTATGTCAGCCTGGG	TAATACGACTCACTATAGGG AAGGCCCGGTTGCAGTG
Vimentin	AAGAGCTCGTCCTACCGCAG	TAATACGACTCACTATAGGG TCGGCCAGGGAGAAGTCC
N-Cadherin	CAGCCGGATACTCTGGAACC	TAATACGACTCACTATAGGG TGCTCCCCACCACTACTTGAA
E-Cadherin	ACCACGAGAATTTTCAATTTGC	TAATACGACTCACTATAGGG CCCAGTGTAACAGCAATGGTG

### Protein isolation and Western blotting

Proteins were harvested at different time points post-amputation and included 1 mm of stump from the amputation site. Samples were sonicated in 50 μL of Laemmli solution containing 200 mM DTT and 50 mM of NaF using the Sonic Dismenbrator Model 100 (Fisher Scientific) for 10 seconds on ice. The proteins were quantified using the EZQ® Protein quantification kit (Thermo Fisher, R33200) following manufacturer’s guidelines. For Western blotting, 20 μg of each sample was used as described in Denis *et al*.^1^. Identical conditions were used for all antibodies. Blocking was done 1 hour at RT in 10% Chicken serum (Thermo Fisher, 16110082) in TBS-T Primary antibodies incubations were done at 4 °C O/N. The p-JNK (Cell Signaling Technology, Phospho-SAPK/JNK (Thr183/Tyr185), 4668)), p-p38 (Cell Signaling Technology, Phospho-p38 MAP Kinase (Thr180/Tyr182), 9211), total p38 (Cell Signaling Technology, p38 MAPK, 9212), p-SMAD2 (Cells Signaling Technology, Phospho-SMAD2, 3108) and total SMAD2 (Cell Signaling Technology, SMAD2, 5339) antibodies were used 1/1000 in (5% BSA, 0.2% Tween20, 0.002% NaN_3_ in 1X TBS). The loading controls: GAPDH (Sigma-Aldrich, Anti-GAPDH, G8795) was used at 1:10000 in blocking solution and alpha-Tubulin at 1:5000 (Neomarkers, alpha-Tubulin, DM1A) in blocking solution. For immuno-detection, we used Lumili-light^Plus^ ECL (Roche, 12015196001) for p-JNK, p-p38, total p38, p-SMAD2 and the 20X LumiGlo^®^ ECL Reagent (Cell Signaling Technology, 7003) for GAPDH, Tubulin and total SMAD2. Revelation was done using auto-radiographic films (Progene, 39–20810).

### Eosin and Hematoxylin coloration

Regenerating limbs were processed as described above for paraffin embedment. Slides were successively put in Mayer’s hematoxylin (Dako Cytomation, S3309) for 75 sec, thoroughly rinsed in water, put in 0.08% NH_4_OH for 20 sec, in 80% ethanol for 1 min, in Eosin for 30 sec and rinsed in 80% ethanol for 30 sec. Samples were dehydrated in 95% ethanol two times for 1 min, 100% twice for 1 minute and two times in xylene for 1 minute. Mounting was done in Permount^TM^ Mounting medium (Fisher Scientific, Sp15-100). Slides were visualised using a Zeiss Axio Imager M2 Optical Microscope (Zeiss, Munich, Germany). Picture were taken with the Axiocam 506 color using tiling. A composite image was made followed by a stitching step and cropped to show the area of interest.

## Supplementary information

Supplementray figures and Western blot scans
