# Author Correction: Epithelial to mesenchymal transition is mediated by both TGF-β canonical and non-canonical signaling during axolotl limb regeneration

**DOI:** 10.1038/s41598-023-28207-w

**Published:** 2023-01-17

**Authors:** Fadi Sader, Jean-François Denis, Hamza Laref, Stéphane Roy

**Affiliations:** 1 Department of Biochemistry and Molecular Medicine, Faculty of Medicine, Université de Montréal, Montréal, Québec Canada; 2 Department of Stomatology, Faculty of Dentistry, Université de Montréal, Montréal, Québec Canada

Correction to: *Scientific Reports* 10.1038/s41598-018-38171-5, published online 04 February 2019

This Article contains an error in Figure 2, where an incorrect image is displayed in panel G'.

The corrected Figure 2 and its accompanying legend appear below.

**Figure 2:**
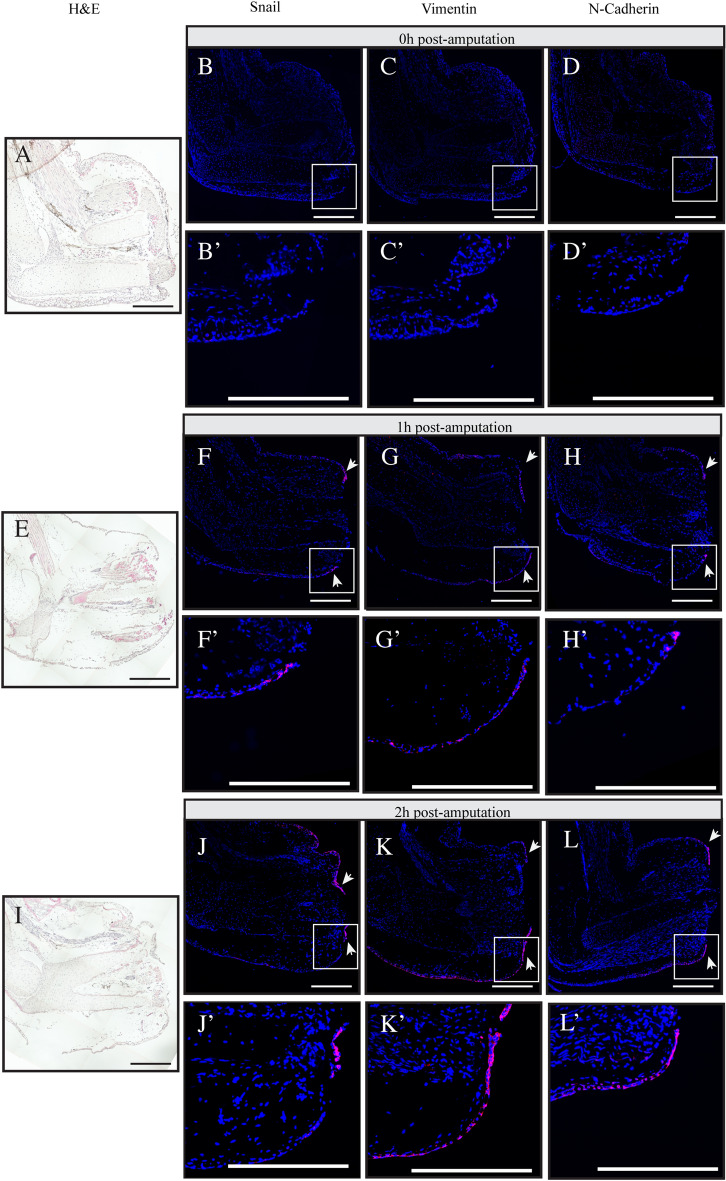
In situ hybridization using tyramide signal amplification showing the expression of EMT markers during regeneration. Different regeneration time points (**A**–**D**) Time 0 h, (**E**,**F**) Time 1 h post-amputation, (**I**–**L**) Time 2 h post-amputation. (**A**,**E**,**I**) Hematoxylin and eosin (H & E) coloration. Overlay of nuclei staining with DAPI (blue) and In situ hybridization with Cy5 (red) for (**B**-**B**’,**F**-**F**’,**J**-**J**’) Snail, (**C**-﻿**C**’,**G**-**G**’,**K**-**K**’) Vimentin, (**D**-**D**’,**H**-﻿**H**’,**L**-**L**’) N-Cadherin. White boxes represent magnified areas. White arrows show the signal in migrating epithelia. Scale bars are 200 μm. Composite images are shown.

