# Supplementary material for: Epithelial to mesenchymal transition is mediated by both TGF-β canonical and non-canonical signaling during axolotl limb regeneration
Source: Sci Rep. 2019 Feb 4;9:1144. doi: 10.1038/s41598-018-38171-5 (PMC6362101; doi:10.1038/s41598-018-38171-5)
Supplement: Supplementary file 1 — Supplementray figures and Western blot scans [file 41598_2018_38171_MOESM1_ESM.pdf]

Supplementary figures and information

Epithelial to mesenchymal transition is mediated by both TGF-β canonical and non-canonical signaling during axolotl limb regeneration

Sader Fadi, Denis Jean-François, Laref Hamza, *Roy Stéphane

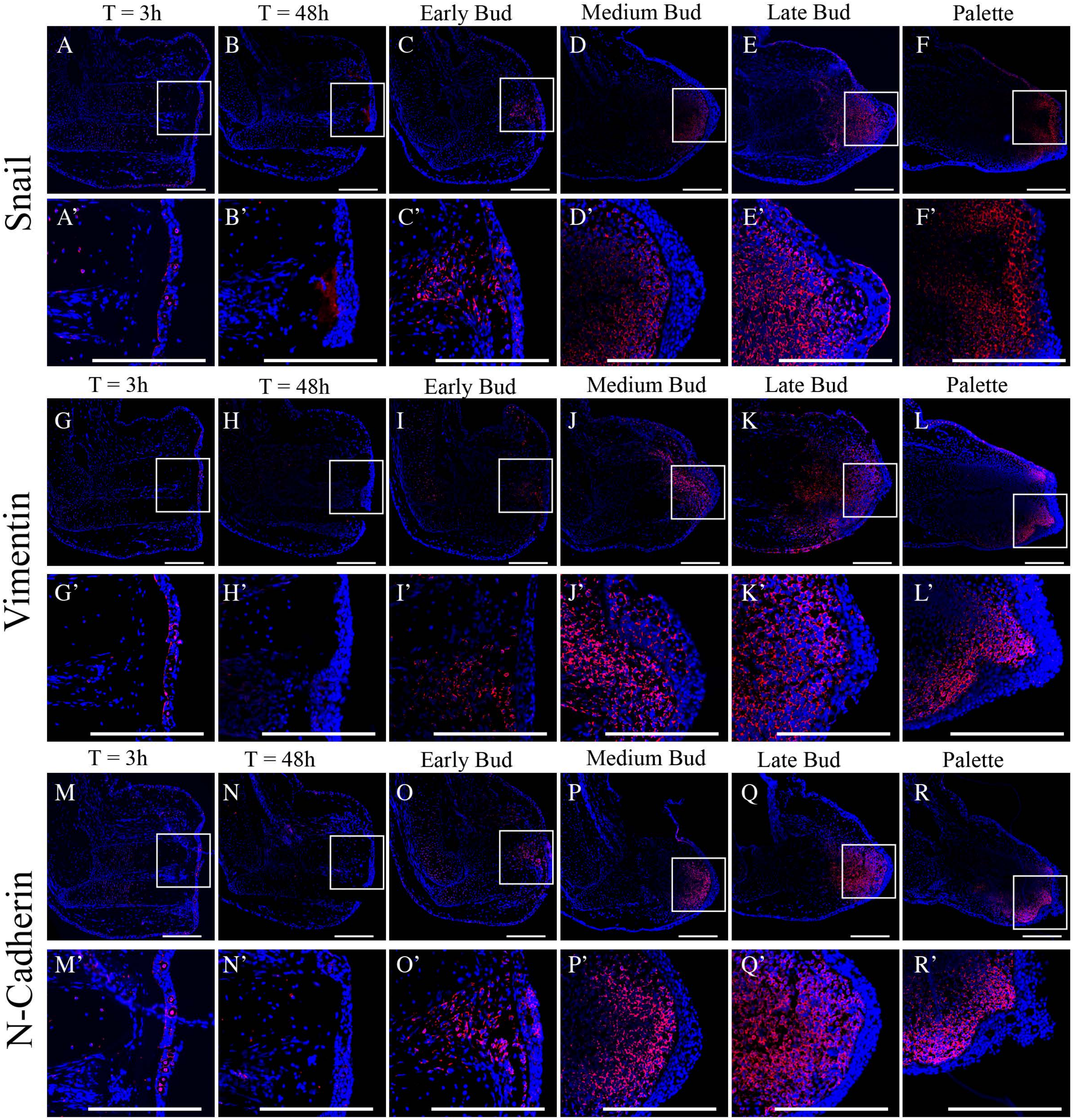

Supplementary Figure 1 In situ hybridization using tyramide showing the expression of Snail, Vimentin and N-Cadherin during regeneration. (A, G, M) 3h post-amputation. (B, H, N) 48h post-amputation. (C, I, O) Early Bud stage. (D, J, P) Medium Bud stage (E, K, Q) Late Bud stage (F, L, R) Palette stage. Overlay of nuclei staining with DAPI (blue) and In situ hybridization with Cy5 (red) for (A-F and A'-F' magnified view of box) Snail; (G-L and G'-L' magnified view of box) Vimentin; and (M-R and M'-R' magnified view of box) N-Cadherin. White boxes represent magnified areas. Scale bars are 200µm. Composite images are shown.

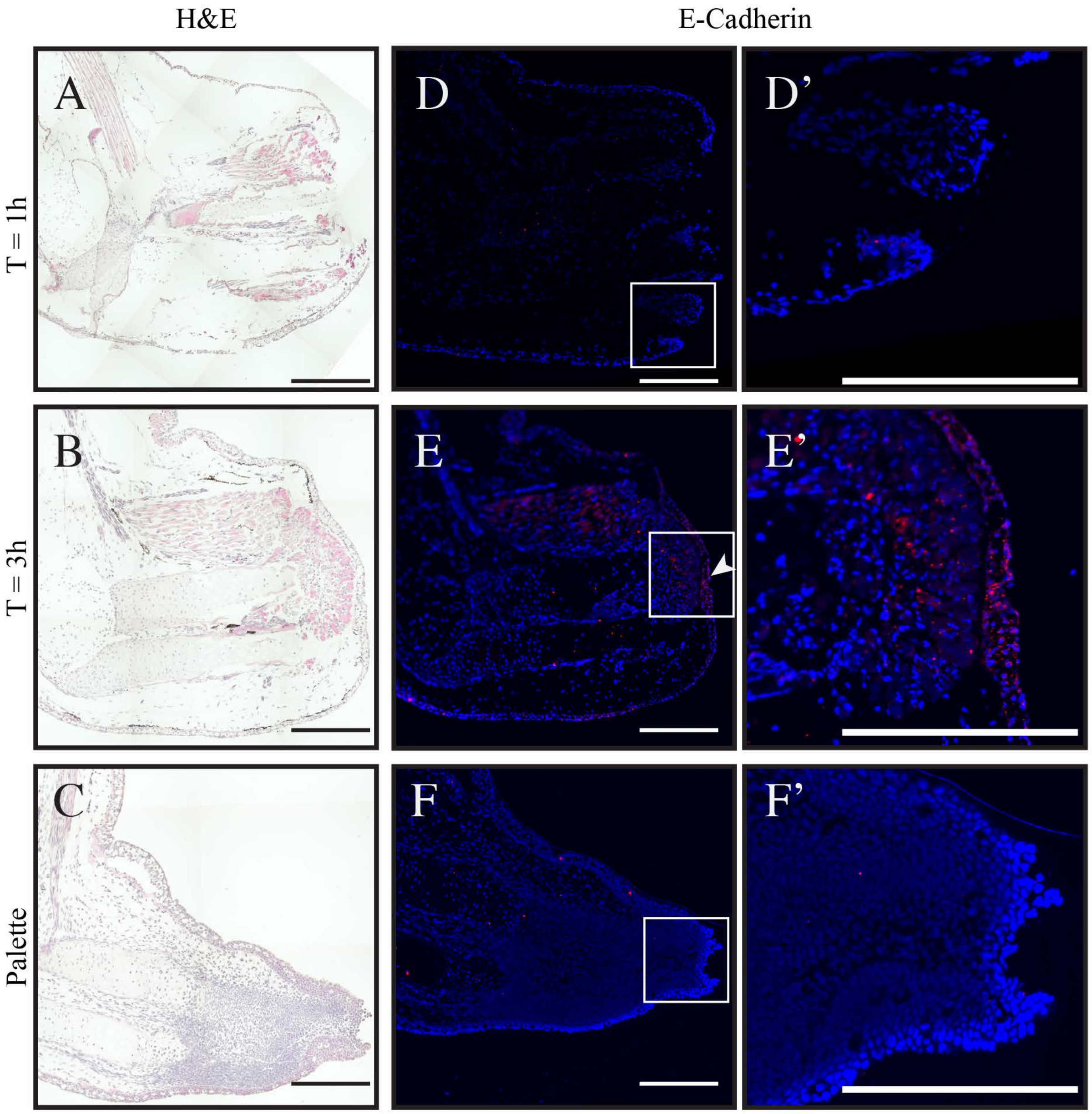

Supplementary Figure 2 In situ hybridization using tyramide showing the expression of E-Cadherin during regeneration. Different regeneration time points (A, D) Time 1h post-amputation, (B, E) Time 2h post-amputation with a closed wound, (C, F) Stage palette. (A-C) Hematoxylin and eosin coloration. (D-F) Overlay of nuclei staining with DAPI (blue) and In situ hybridization with Cy5 (red) for E-Cadherin (D'-F' magnified view of box). White boxes represent magnified areas. White arrows show the signal in recently formed epithelia. Scale bars are 200µm. Composite images are shown.

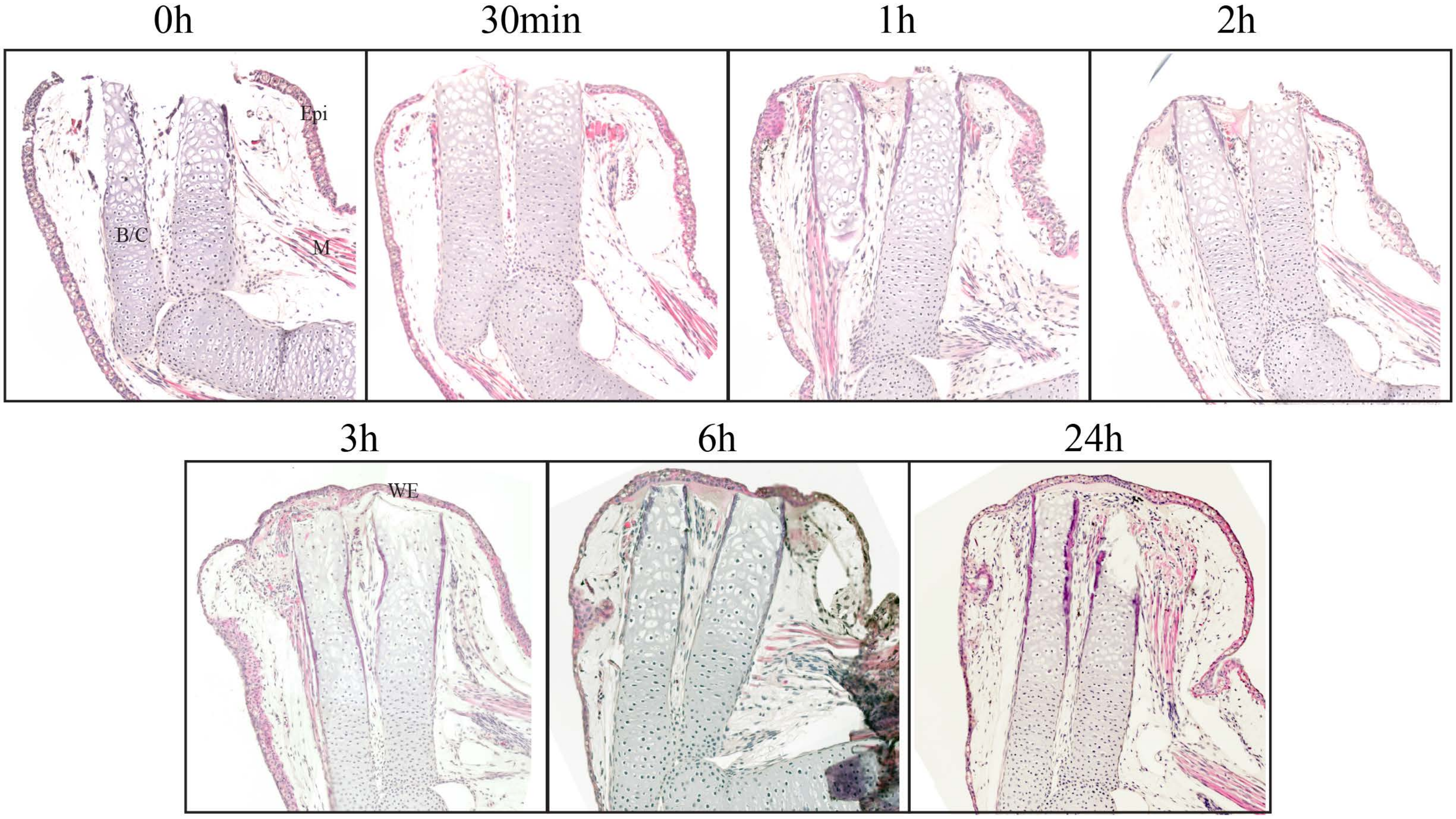

Supplementary Figure 3 Time course of normal wound closure following limb amputation in axolotls. Hematoxylin and Eosin Staining showing time points from 0h (Animals are fixed after amputation) to 24h. Within 3h wound is closed and the epidermis has covered the injury site and formed the wound epidemis. Animals are between 4 and 5 cm. (WE) wound epidermis, (M) Muscle, (Epi) Epidermis, (B/C) Bone and cartilage.

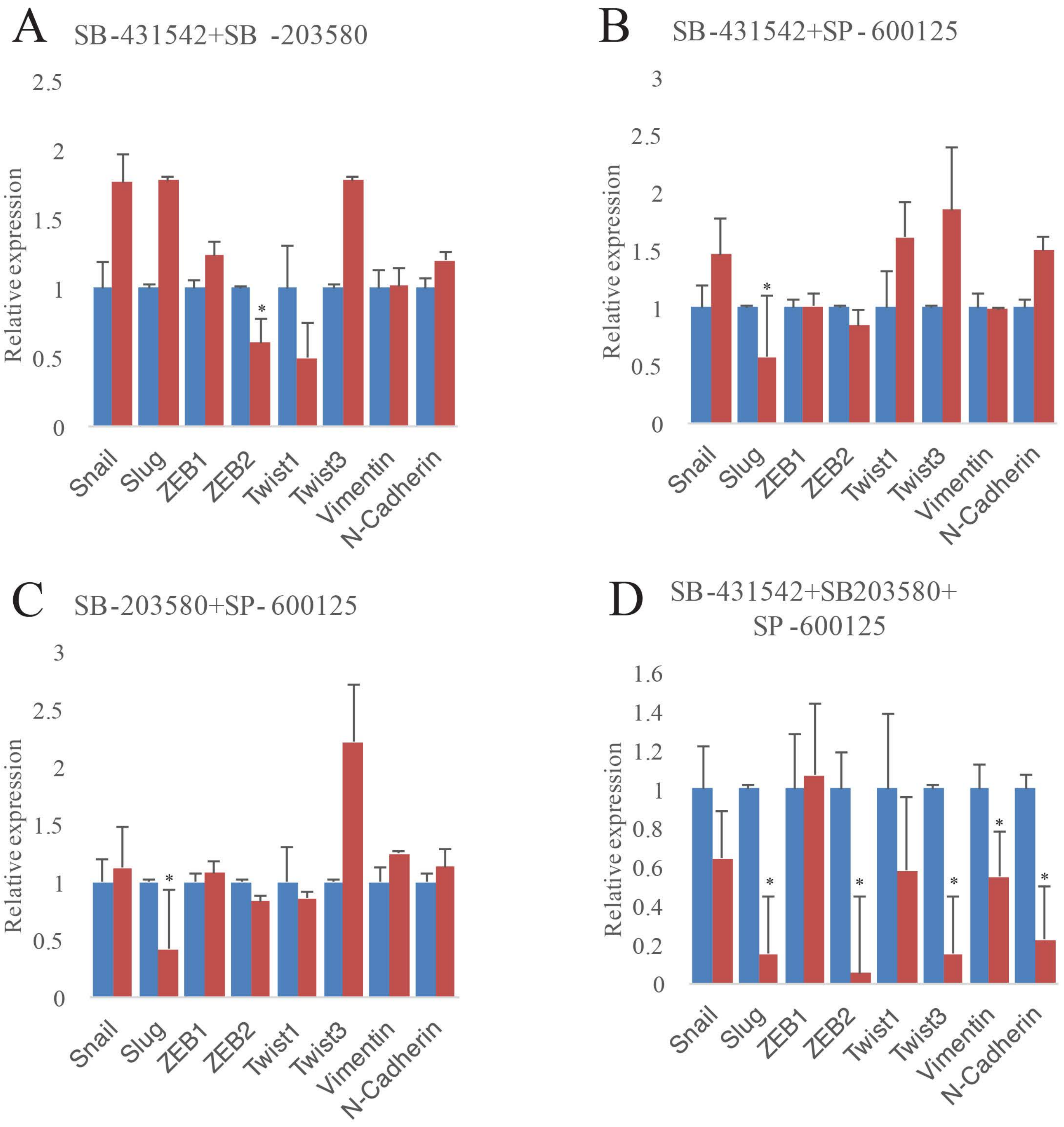

Supplementary Figure 4 RT-qPCR for EMT markers on axolotl limb for animal treated with different combinations of SB-431542, SB-203580 and SP-600125. Animals were treated 24h before amputation and 1h post-amputation before harvesting. Blue bars are DMSO controls and red bars are for animals treated with; (A) SB-431542 and SB-203580, (B) SB-431542 and SP-600125, (C) SB-203580 and SP-600125 and (D) SB-431542, SB-203580 and SP-600125. Using Excel, a two-tailed Student's t test was performed to compare DMSO controls to the different treatments *$p < 0.05$, Means±s.e.m. (normalized using GAPDH), N = 4.

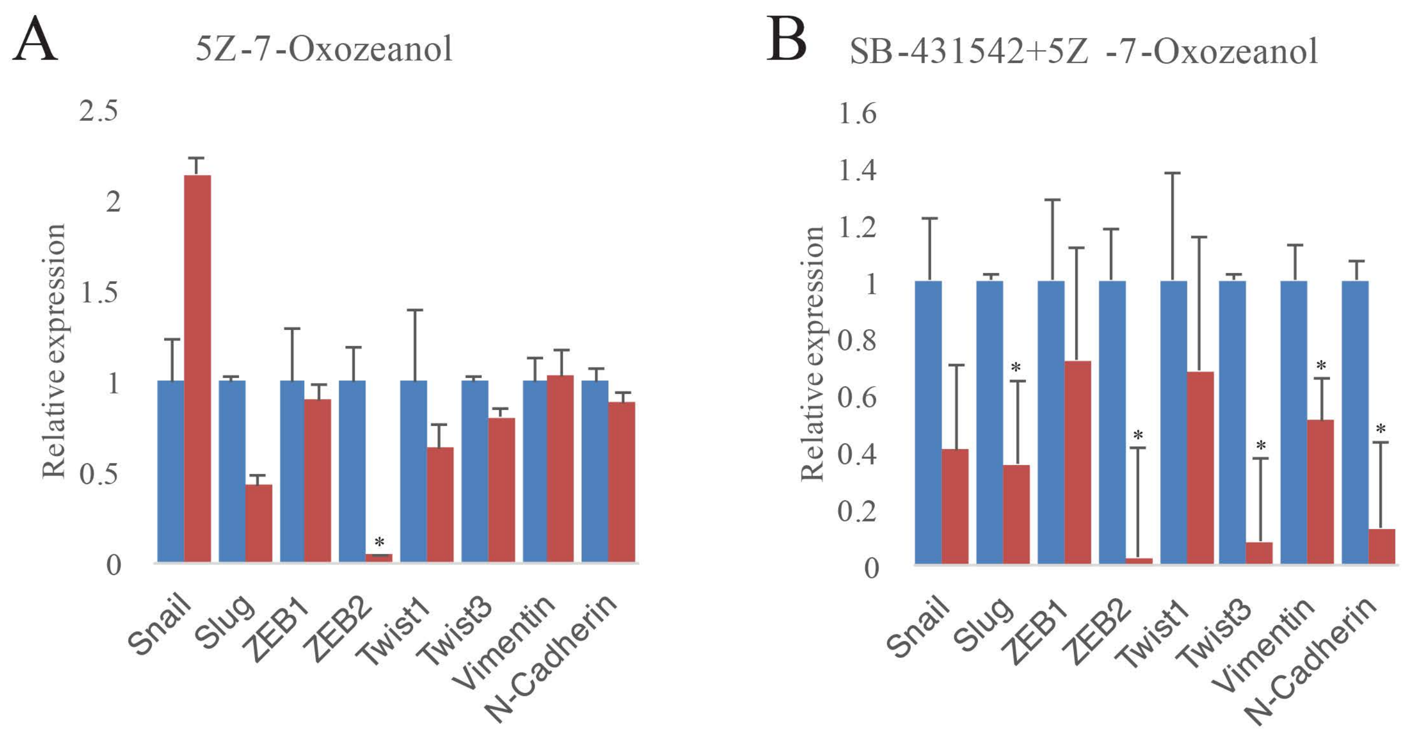

Supplementary Figure 5 RT-qPCR for EMT markers on axolotl limb for animal treated with combination of 5Z-7-Oxozeanol and SB-431542. Animals were treated 24h before amputation and 1h post-amputation before harvesting. Blue bars are DMSO controls and red bars are for animals treated with; (A) 5Z-7-Oxozeanol, (B) SB-431542 and 5Z-7-Oxozeanol. Using Excel, a two-tailed Student's t test was performed to compare DMSO controls to the different treatments *p <0.05, Means±s.e.m. (normalized using GAPDH), N = 4

Western Blot Scans Corresponding to Figure 3

60kD
50kD

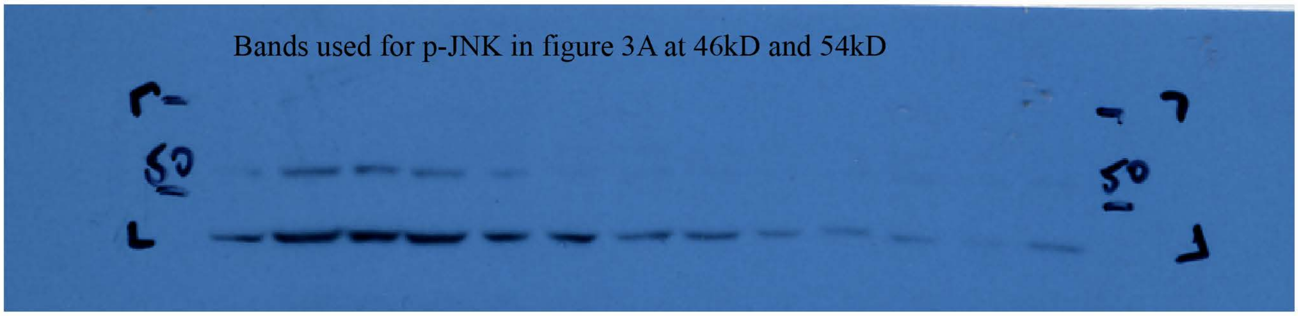

40kD
30kD

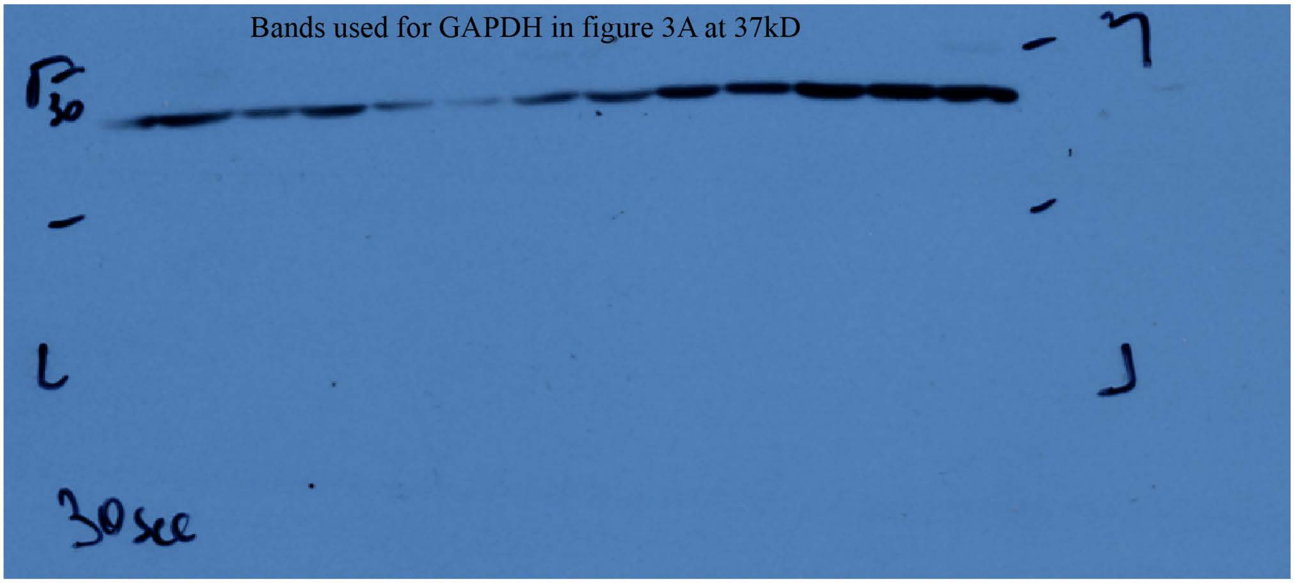

40k
30k

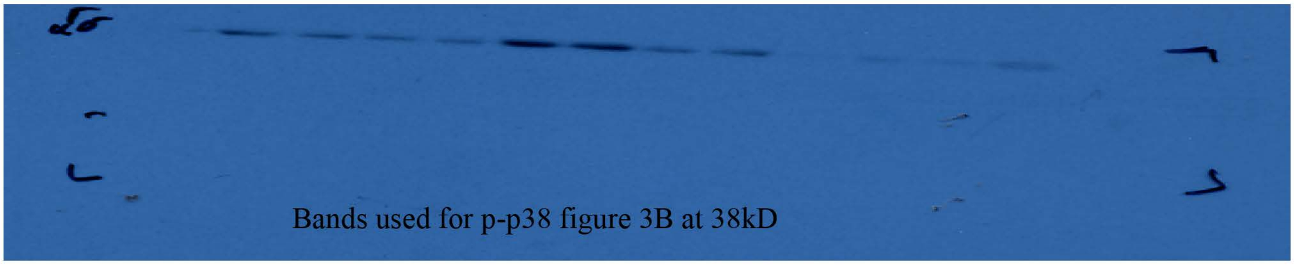

40kD
30kD

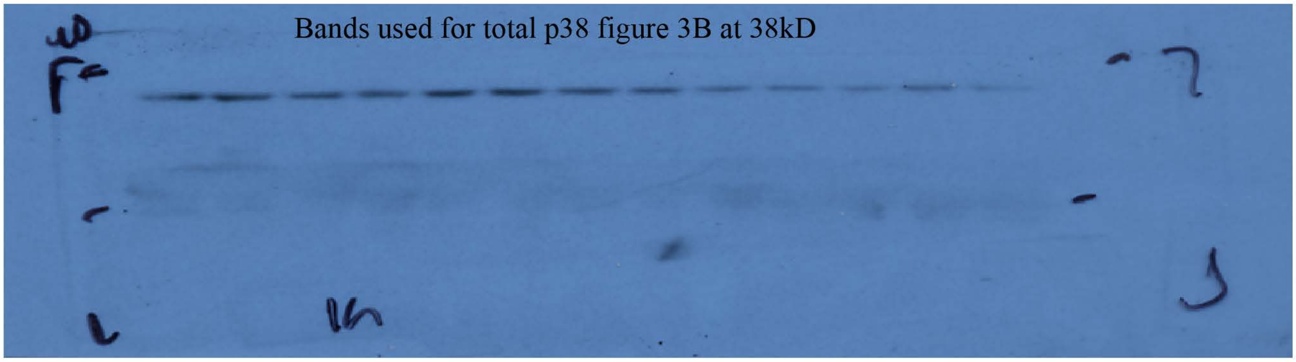

40kD
30kD

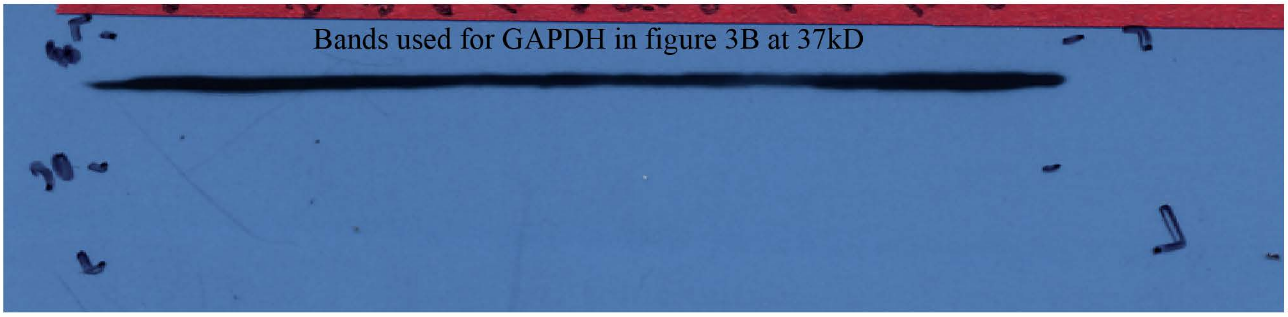

Western Blot Scans Corresponding to Figure 4

60kD

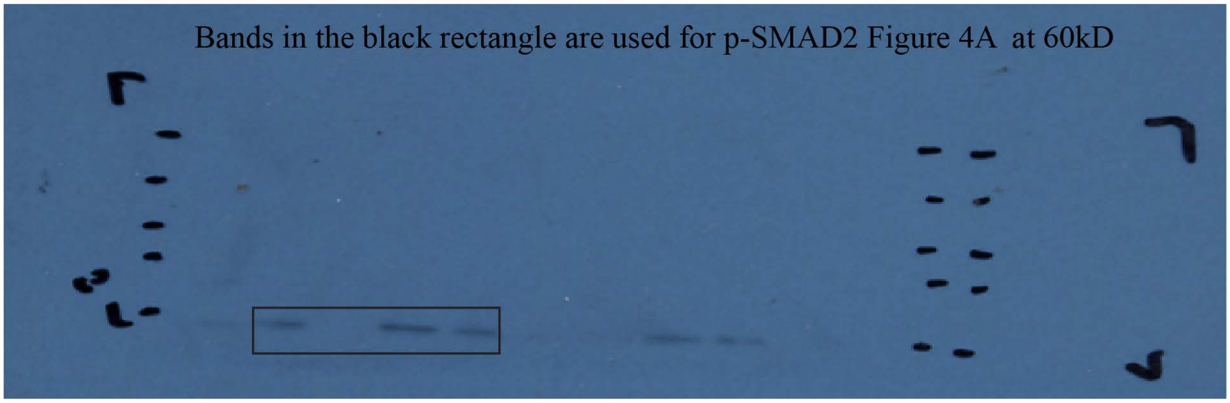

60kD

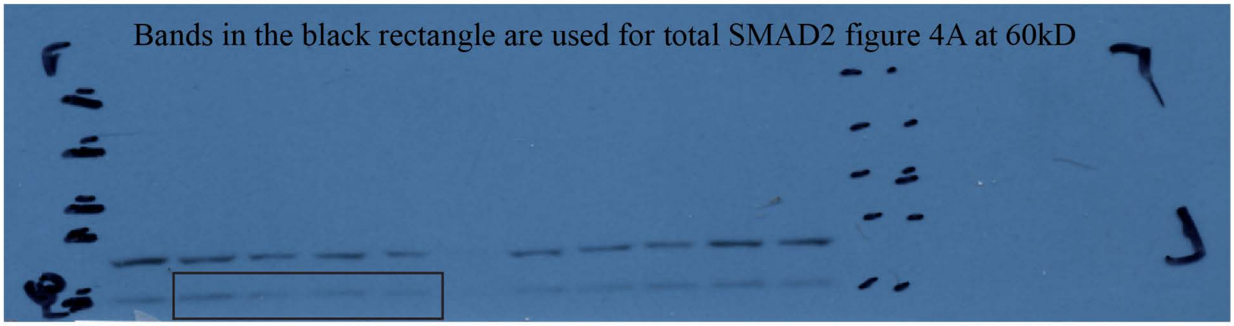

40kD

30kD

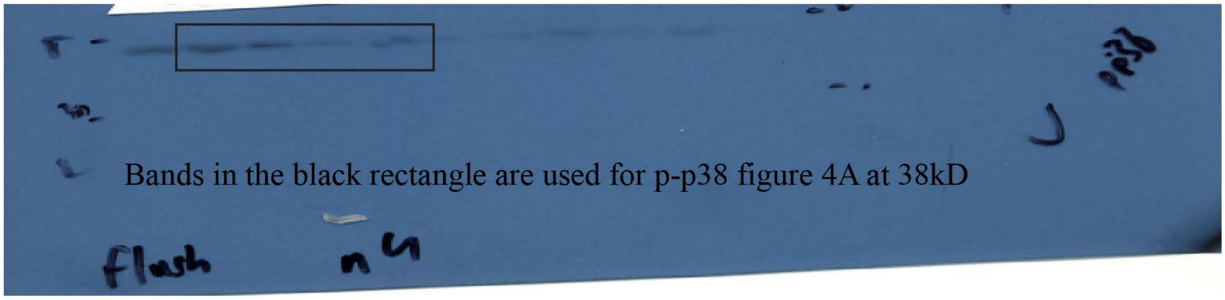

50kD

40kD

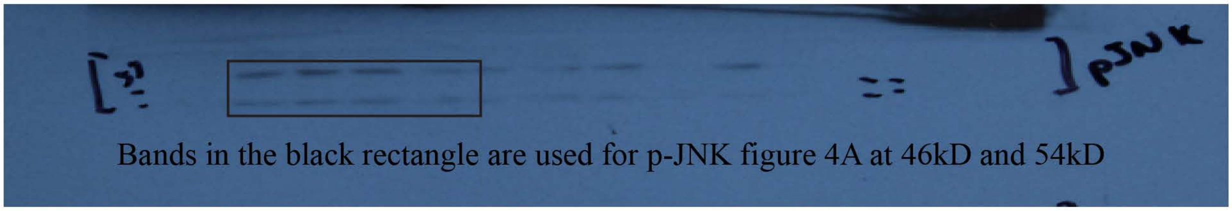

50kD

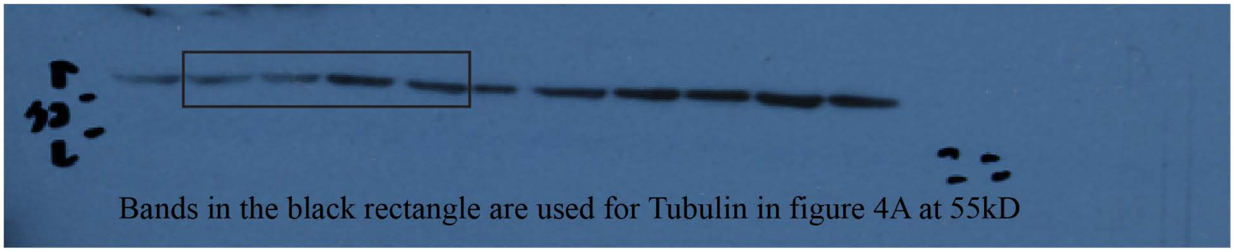

Western Blot Scans Corresponding to Figure 6

60kD

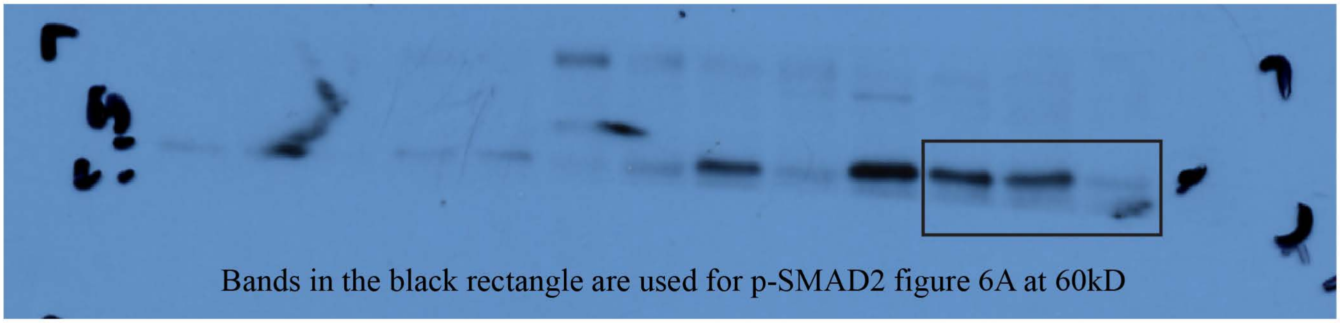

Bands in the black rectangle are used for p-SMAD2 figure 6A at 60kD

60kD

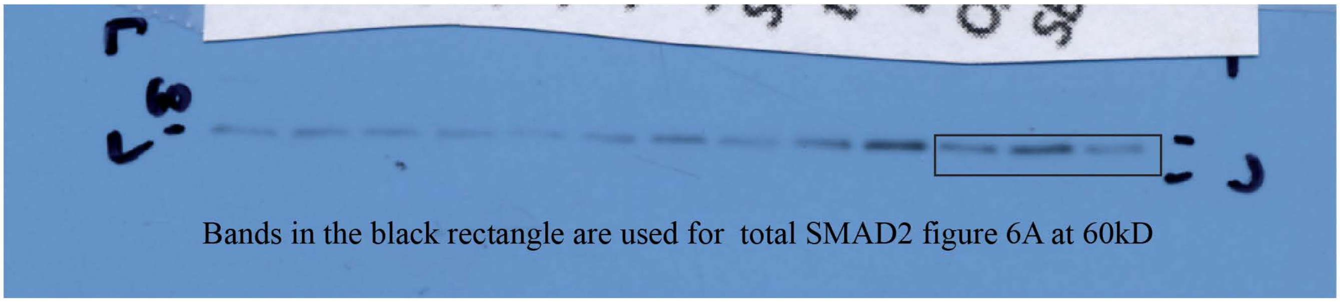

Bands in the black rectangle are used for total SMAD2 figure 6A at 60kD

50kD

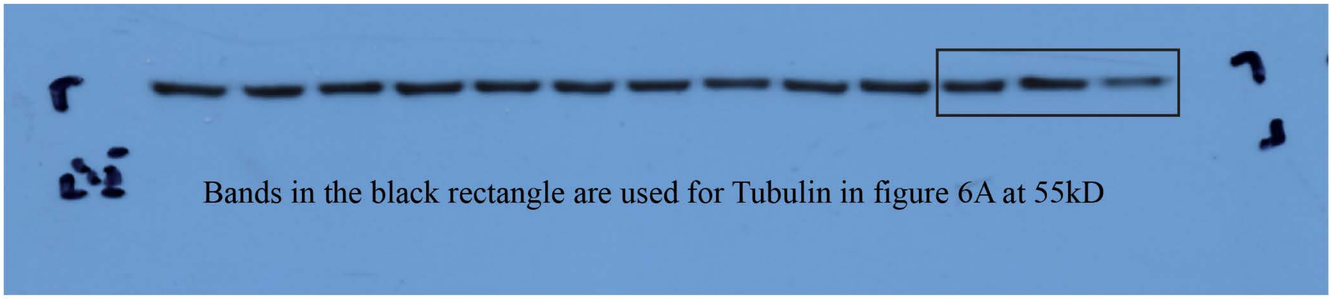

Bands in the black rectangle are used for Tubulin in figure 6A at 55kD

40kD

30kD

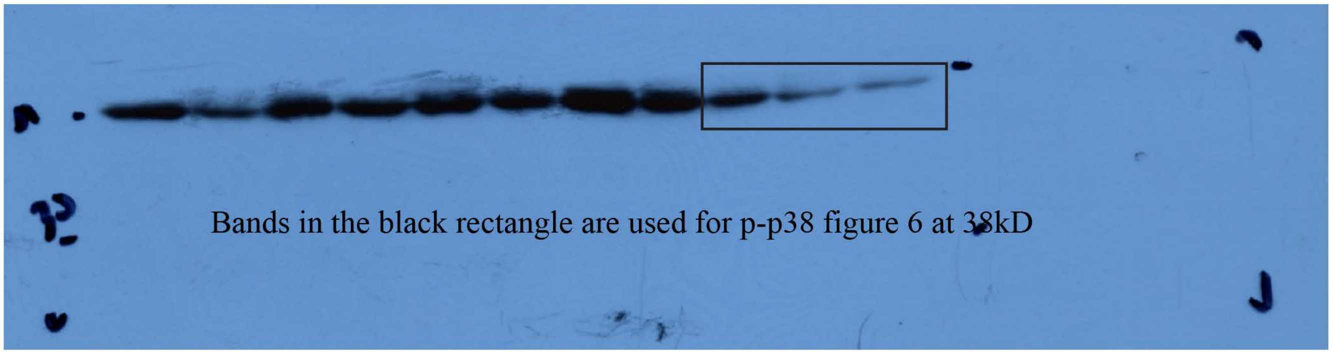

Bands in the black rectangle are used for p-p38 figure 6 at 38kD

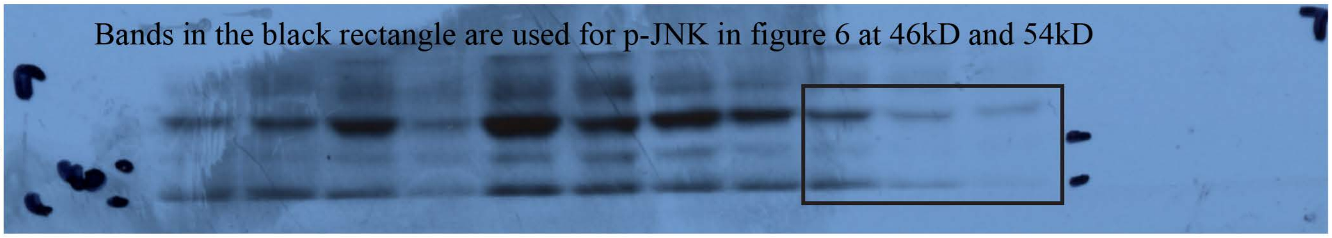

Bands in the black rectangle are used for p-JNK in figure 6 at 46kD and 54kD

50kD

40kD

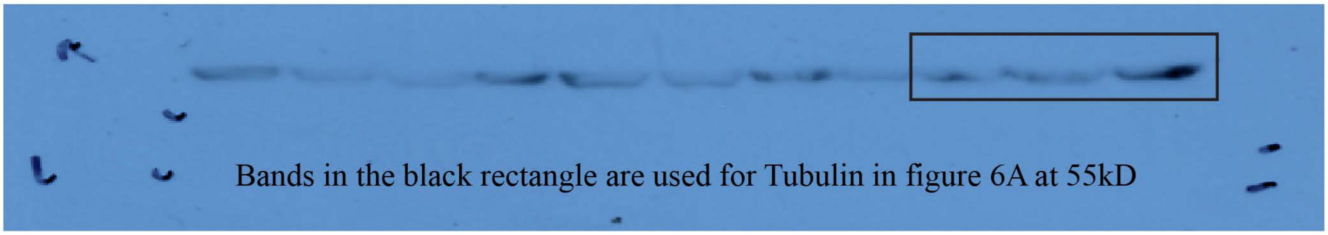

Bands in the black rectangle are used for Tubulin in figure 6A at 55kD

50kD

40kD
